# Childhood Maltreatment, Stressful Life Events, Cognitive Emotion Regulation Strategies, and Non-suicidal Self-Injury in Adolescents and Young Adults With First-Episode Depressive Disorder: Direct and Indirect Pathways

**DOI:** 10.3389/fpsyt.2022.838693

**Published:** 2022-04-12

**Authors:** Hong Qian, Chang Shu, Li Feng, Junyi Xiang, Ying Guo, Gaohua Wang

**Affiliations:** ^1^Department of Psychiatry, Renmin Hospital of Wuhan University, Wuhan, China; ^2^Wuhan Mental Health Center, Tongji Medical College, Huazhong University of Science and Technology, Wuhan, China

**Keywords:** childhood maltreatment, stressful life events, cognitive emotion regulation strategies, non-suicidal self-injury, major depressive disorder

## Abstract

**Introduction:**

Childhood maltreatment (CM), stressful life events (SLE), and cognitive emotion regulation strategies (CERS) have been considered crucial in the development of non-suicidal self-injury (NSSI) and major depressive disorder (MDD), but the pathways of this association are not clear. We aim to identify direct effects of CM and SLE on NSSI and depression severity and its indirect effects *via* CERS in adolescents and young adults with a diagnosis of MDD.

**Methods:**

A total of 114 patients (aged 14–24 years) with first episode MDD were included and further divided into the NSSI group (*n* = 56) and non-NSSI group (*n* = 58) according to the DSM-5 criteria. Diagnostic interviews and self-report measures were conducted to assess CM, SLE, CERS, and diagnose NSSI. Severity of depressive symptoms was measured using the Hamilton Rating Scale (HAMD). The structural equation model was used to assess the pathways.

**Results:**

MDD patients with NSSI had more frequent family history of mental illness, more experience of CM and SLE, more serious depression, less use of adaptive CERS, and more use of maladaptive CERS. In the final structural equation model (χ2 = 4.82, df = 6, *p* = 0.57, CFI = 1.0, TLI = 1.10, and RMSEA = 0), the experience of CM and SLE showed a significant indirect effect on NSSI through adaptive CERS. CM and SLE only had direct effects on depression severity.

**Conclusions:**

NSSI are prevalent in adolescents and young adults with MDD and highly intertwined with CM, SLE, and CERS. Adaptive CERS, not maladaptive CERS may be a possible mechanism relating CM and SLE to NSSI in MDD patients.

## Introduction

Non-suicidal self-injury (NSSI), defined as the behavior of harming one's own body without suicidal intent, is highly prevalent in adolescents and young adults (1). It is reported that the prevalence of NSSI is 17.2% in adolescents and 13.4% in young adults, which are greatly higher than that in adults (5.5%) (1). Furthermore, frequent engagement in NSSI has been an important risk factor for suicidal behaviors among adolescents and adults in both community population and psychiatric patients (2). Major depressive disorder (MDD) is a prevalent psychiatric disorder, which often accompanies NSSI, and the presence of both may result in a high risk of suicidality (3). Depressed adolescents have shown greater and more stable NSSI engagement than adolescents without depression (4). Despite suicide being demonstrated in several studies as a result of a complex interaction of genetic, neurobiological, environmental, and psychological factors, the precise pathophysiological mechanisms are not yet fully understood, and clinical interventions may not be efficacious, particularly among MDD subjects (5, 6). Therefore, the problem of NSSI in adolescents and young adults has been widely of concern, especially among depression patients.

Stressors, either stressful life events around the time of symptom onset, or historical adversity, particularly childhood maltreatment, have been considered key factors for the cause of depression and NSSI (7–10). Childhood maltreatment, which involves the aspects of childhood abuse and neglect is found to be associated with NSSI in MDD (7, 8). Stressful life events (SLE), which cover many aspects of stress (such as interpersonal, academic stress, health adaptation, and other dimensions), are closely related to NSSI; current studies find that self-injurers experience significantly greater accumulation of stressful life events compared with non-self-injurers (11), and stressful life events can predict depression and self-harm in adolescents (10, 12). Literature suggests that childhood adversities and recent life events may have cumulative effects. For example, among psychotic patients, the effect of recent stressful life events on psychosis may be amplified by exposure to childhood maltreatment and the co-occurrence of recent life events, and childhood maltreatment can increase the risk of developing psychosis (13, 14). Those who experienced childhood maltreatment were more likely to develop depression triggered by stressful life events (SLE), which is called stress sensitization that has been observed in adolescents (15) and adults (16). Heightened sensitivity to stimuli and emotional responses to threats, difficulties with emotion regulation have all been presented as potential mechanisms of stress sensitization (17). In recent years, with the deepening studies on the association between childhood maltreatment and NSSI, studies have found that alexithymia and self-blame play a partially mediating role (18, 19). Studies also show that MDD is associated with impaired emotion regulation (20), which may explain the relationship between childhood environment and NSSI in MDD patients. Despite studies implicating that a history of maltreatment and deficits in emotion regulation strategies are contributors to NSSI and depression severity, much is still unknown about the specificity pathway from both childhood maltreatment and stressful life events to NSSI and depression severity.

Penelope Hasking has summarized four models of emotion regulation and provided the cognition–emotion model theory to explain and predict NSSI in individuals (21). Cognitive emotion regulation strategies (CERS) are defined as adaptive and maladaptive cognitive strategies to regulate emotional experiences. Existing studies have suggested that emotion dysregulation is associated with numerous psychiatric difficulties and risky behaviors (22), and it is well established that poor emotion regulation is a core process underlying NSSI (23). Recently, there have been some studies examining the presence of childhood maltreatment along with more maladaptive or fewer adaptive emotion regulation strategies that may develop NSSI (7, 19) and depression (24). Stressful life events also have been found to be associated with NSSI (11) and depression (25, 26) through cognitive emotion regulation strategies. However, little studies were focused on the interrelations of both childhood maltreatment and stressful life events in NSSI and the underlying mechanisms of the pathway through emotion regulation strategies.

**Figure 1:**
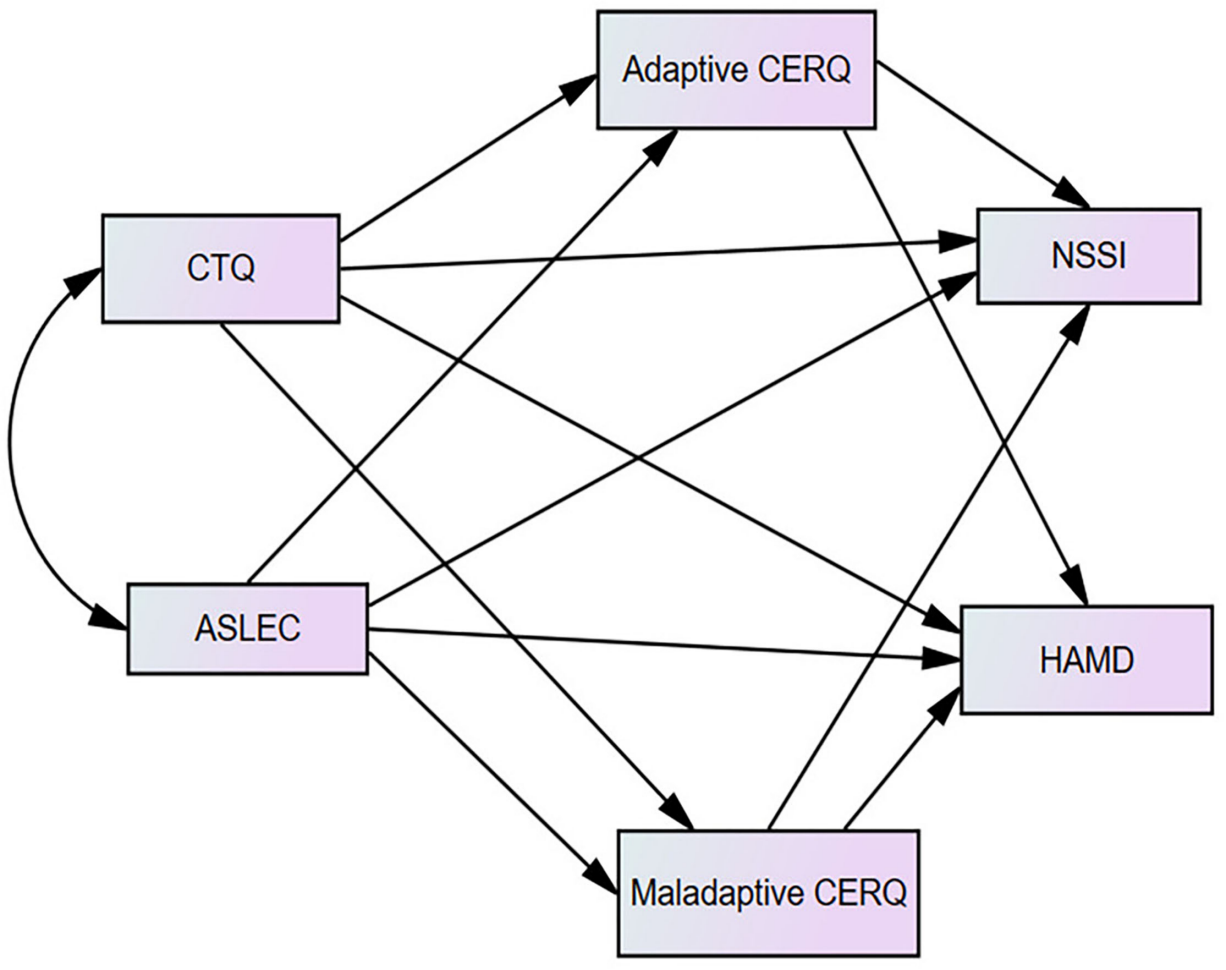
The hypothetical model of the mediating role of adaptive and maladaptive cognitive emotion regulation questionnaire (CERQ).

To our knowledge, this is the first study identifying the pathway from childhood maltreatment and stressful life events to NSSI and depression severity through cognitive emotion regulation strategies in adolescents and young adults with first episode MDD. According to the mentioned literature, our research aims to investigate clinical features of MDD patients with NSSI and an underlying mechanism of how childhood maltreatment and stressful life events affect NSSI and depression severity, in order to provide clinically useful evidence for the effective prevention and intervention for NSSI and depression. Our hypothetical model in this study is as in Figure 1.

## Materials and Methods

### Participants

A total of 114 youth participants (ages from 14–24) with first episode MDD at Renmin Hospital of Wuhan University from July 2020 to July 2021 were recruited. All participants were diagnosed with MDD by two experienced psychiatrists following the Diagnostic and Statistical Manual of Mental Disorders (5th ed.) (DSM-5) (27, 28). The Mini International Neuropsychiatric Interview, Version 5.0 (MINI) was used to screen for current mental disorders, including MDD (29). The inclusion criteria were in the first episode of MDD, had never taken antidepressants, and aged from 14 to 24. The exclusion criteria were other mental disease (active psychotic symptoms or mania, developmental disorder, substance abuse), any use of psychoactive drug, any history of neurological disease, and severe physical disease. Finally, there were 114 patients included in our study. According to the diagnose criteria of NSSI recommended by DSM-5, the enrolled patients were further divided into the NSSI group (labeled 1) and the non-NSSI group (labeled 2). All patients provided informed consent and were guaranteed anonymity. When patients were younger than 18 years, parental approval for patients was requested.

### Depressive Symptom Severity

The Hamilton Rating Scale (HAMD) (30) was used to assess the severity of depression. Psychiatrists assessed symptoms in patients with MDD using the HAMD-17, a five-point scale of 0 to 4. The total score can reflect the severity of the disease, that is, the more severe the illness, the higher the total score.

### Childhood Maltreatment

The Childhood Trauma Questionnaire (CTQ) was used to assess the childhood maltreatment experience (31). It had 28 items, rated on a five-point Likert scale from 1 (never true) to 5 (very often true), which was divided into five subscales: emotional abuse (no <13 is positive), physical abuse (no <10 is positive), sexual abuse (no <8 is positive), emotional neglect (no <15 is positive), and physical neglect (no <10 is positive).

### Stressful Life Event Assessment

The occurrence and severity of stressful life events in adolescents in the past 12 months were assessed by the Adolescent Self-Rating Life Events Checklist (ASLEC) (25, 32), which included 27 items and was divided into six factors: relationship pressure, learning pressure, being punished, loss, adaptation problem, and “other.” If the events did not occur, the score was 0; if the events did occur, they were selected according to the impact of the events from 1 (no effect) to 5 (very severe). The higher the score was, the more serious was the impact of stressful life events.

### Cognitive Emotion Regulation Strategies

The Cognitive Emotion Regulation Questionnaire (CERQ) (33) was used to assess cognitive emotion regulation strategies, which participants used in response to the experience of threatening or stressful life events. The scale involves 36 items consisting of nine strategy subscales to assess adaptive (acceptance, positive refocusing, refocus on planning, positive reappraisal, and putting into perspective) and maladaptive (self-blame, rumination, catastrophizing, and blaming others) CERS. Cognitive emotion regulation strategies were rated on a five-point Likert scale from 1 (never) to 5 (always).

### Data Analysis

Continuous variables with normal distributions were expressed as mean and standard deviation. Categorical variables were expressed as number and percentage (%). Student's *t*-tests (normally distributed) or Mann–Whitney U-test (non-normally distributed) were used for comparison with continuous variables between MDD patients with and without NSSI. Chi-square tests were used to analyze the comparison with categorical variables. Pearson's correlation analysis was used to explore the relationship between CM, SLE, adaptive and maladaptive CERS, NSSI, and depression severity.

We applied the structural equation modeling (SEM) to assess the direct and indirect effects of CM and SLE on NSSI and depression severity through CERS (Figure 1). In the model, CM and SLE were independent variables, NSSI and depression severity were dependent variables, adaptive and maladaptive CERS were mediators. A well-fitting SEM is indicated when Tucker–Lewis index (TLI) and comparative fit index (CFI) is at least 0.90, and the root mean square error of approximation (RMSEA) is 0.06 or lower. Finally, we performed bootstrapping with 1,000 resamples to examine the significance of the mediation effect. All the above statistical analyses were performed using SPSS Statistics (Version 22.0, IBM, Armonk, NY, USA) and AMOS 26.0 software (AMOS, Chicago, IL, USA). The *p*-value was two sided, and an alpha <0.05 was considered to define statistical significance.

## Results

### Demographic and Clinical Characteristics

Demographic and clinical characteristics are summarized in Table 1. A total of 114 youth depression patients participated (88 females, 77.2%; mean age = 17.28, SD = 2.96). Fifty-six (49.1%) had been diagnosed with NSSI. Moderate to severe depression was also observed with a mean HAMD scores of 21.56 (±5.28). Moreover, the mean CTQ score was 55.03 (±16.62), including emotional abuse (12.15 ± 5.06, 38.6% positive), physical abuse (7.54 ± 3.64, 26% positive), sexual abuse (6.07 ± 2.42, 16% positive), emotional neglect (14.70 ± 5.37, 63% positive), and physical neglect (9.62 ± 3.95, 50% positive).

**Table 1:** Demographic and clinical characteristics of patients with MDD (*n* = 114).

**Characteristics**	**Mean ±SD or *n* (%)**
Age, years	17.28 ± 2.96
Gender (female)	88 (77.2%)
**Family structure**	
Two biological parents	84 (73.7%)
One biological parents	30 (26.3%)
Unmarried	114 (100%)
In education	114 (100%)
Family history of mental illness	19 (16.7%)
Non-suicidal self-injury (NSSI)	56 (49.1%)
HAMD	21.56 ± 5.28
CTQ	50.09 ± 15.37
CTQ emotional abuse	12.15 ± 5.06
CTQ emotional neglect	14.70 ± 5.37
CTQ physical abuse	7.54 ± 3.64
CTQ physical neglect	9.62 ± 3.95
CTQ sexual abuse	6.07 ± 2.42
ASLEC	48.53 ± 21.56
ASLEC relationship pressure	10.47 ± 4.86
ASLEC learning pressure	10.33 ± 5.01
ASLEC being punished	7.32 ± 7.22
ASLEC loss	7.52 ± 5.45
ASLEC adaptation	10.47 ± 4.49
ASLEC other	2.41 ± 1.65
**CERQ**	
CERQ self-blame	14.96 ± 2.45
CERQ acceptance	13.99 ± 3.10
CERQ rumination	14.87 ± 2.80
CERQ positive refocusing	10.39 ± 3.33
CERQ refocus on planning	12.51 ± 3.50
CERQ positive reappraisal	10.68 ± 3.41
CERQ putting into perspective	11.15 ± 2.07
CERQ catastrophizing	12.32 ± 3.30
CERQ blaming others	11.31 ± 3.08
Maladaptive CERQ	53.42 ± 7.55
Adaptive CERQ	58.71 ± 9.81

*HAMD, Hamilton Rating Scale; CTQ, Childhood Trauma Questionnaire; ASLEC, Adolescent Self-Rating Life Events Checklist; CERQ, Cognitive Emotion Regulation Questionnaire; MDD, major depressive disorder*.

### Differences Between Major Depressive Disorder Patients With and Without Non-suicidal Self-Injury

Compared with MDD patients without NSSI, more frequent family history of mental illness (χ^2^ = 11.23, *p* < 0.01), more experience of CM (*t* = 4.59, *p* < 0.01) and SLE (*t* = 4.16, *p* < 0.01), more serious depression (HAMD: *t* = 2.57, *p* < 0.05), less use of adaptive CERS (Mann–Whitney *U*-test, z = 3.40, *p* < 0.01), and more use of maladaptive CERS (*t* = 2.80, *p* < 0.01) were reported in MDD patients with NSSI (Table 2).

**Table 2:** Differences between MDD patients with and without NSSI.

	**MDD with NSSI (*n =* 56)**	**MDD without NSSI (*n =* 58)**		
	***n* (%)**	***n* (%)**	**χ^2^**	***p*-value**
Female	46 (82.1%)	42 (72.4%)	1.53	0.27
Family structure (one biological parent)	14 (25.0%)	16 (28.6%)	0.10	0.83
Family history of mental illness	16 (28.6%)	3 (5.2%)	11.23	<0.01^**^
	**Mean** **±S.D**	**Mean** **±S.D**	**t/z**	***p***
Age	16.96 ± 2.91	17.53 ± 3.14	−1.0	0.32
HAMD	22.82 ± 5.51	20.34 ± 4.77	2.57	0.01^*^
CTQ	56.32 ± 16.54	44.07 ± 11.37	4.59	<0.01^**^
ASLEC	56.52 ± 21.97	40.81 ± 18.23	4.16	<0.01^**^
Maladaptive CERQ	55.38 ± 7.72	51.53 ± 6.94	2.80	<0.01^**^
Adaptive CERQ	55.23 ± 9.73	62.07 ± 8.72	3.40	<0.01^**^

*NSSI, non-suicidal self-injury; HAMD, Hamilton Rating Scale; CTQ, Childhood Trauma Questionnaire; ASLEC, Adolescent Self-Rating Life Events Checklist; CERQ, Cognitive Emotion Regulation Questionnaire.*

* *p < 0.05.*

** *p < 0.01*.

### Correlation Between Childhood Maltreatment, Stressful Life Events, Cognitive Emotion Regulation Strategies, Non-suicidal Self-Injury, and Depression Severity

Based on the scores of CTQ, ASLEC, HAMD, CERQ, and diagnosis of NSSI, adaptive and maladaptive CERS were significantly associated with CM (adaptive CERS: *r* = −0.42, *p* < 0.01; maladaptive CERS: *r* = 0.23, *p* < 0.05), SLE (adaptive CERS: *r* = −0.41, *p* < 0.01; maladaptive CERS: *r* = 0.42, *p* < 0.01), the depression severity (adaptive CERS: *r* = −0.32, *p* < 0.01; maladaptive CERS: *r* = 0.36, *p* < 0.01), and NSSI (adaptive CERS: *r* = 0.35, *p* < 0.01; maladaptive CERS: *r* = −0.26, *p* < 0.01) (Table 3). Results satisfied the requirements for testing mediational effects of CERS.

**Table 3:** Correlation between childhood maltreatment, stressful life event, cognitive emotion regulation strategies, and depression.

	**1**	**2**	**3**	**4**	**5**
1. NSSI	-	-	-	-	-
2. HAMD	−0.24^*^	-	-	-	-
3. CTQ	−0.24^*^	0.44^**^	-	-	-
4. ASLEC	−0.37^**^	0.52^**^	0.58^**^	-	-
5. Maladaptive CERQ	−0.26^**^	0.36^**^	0.23^*^	0.42^**^	-
6. Adaptive CERQ	0.35^**^	−0.32^**^	−0.42^**^	−0.41^**^	−0.17

*Associations between dichotomous and continuous variables are point biserial correlations.*

*NSSI, non-suicidal self-injury; HAMD, Hamilton Rating Scale; CTQ, Childhood Trauma Questionnaire; ASLEC, Adolescent Self-Rating Life Events Checklist; CERQ, Cognitive Emotion Regulation Questionnaire.*

* *p < 0.05.*

** *p < 0.01*.

### Multiple Mediating Effects of Cognitive Emotion Regulation Strategies on the Relationship of Childhood Maltreatment, Stressful Life Events, Non-suicidal Self-Injury, and Depression Severity

According to hypothetical and final model of the mediating role of CERS, we assigned the regression weights for the direct effects among variables (Table 4). For the hypothetical model, an acceptable fitness was shown (χ^2^ = 0.145, df = 2, *p* = 0.93; CFI = 1.0; TLI = 1.10, and RMSEA = 0). After removing non-significant pathways, the final model was obtained, and the final model was effectively fitted (χ^2^ = 4.82, df = 6, *p* = 0.57, CFI = 1.0, TLI = 1.10, and RMSEA = 0).

**Table 4:** Coefficients for direct relations among childhood maltreatment, stressful life event, cognitive emotion regulation strategies, NSSI, and depression in the structural equation model.

**Paths**	**Primary model**		**Final model^a^**	
	**Standardized** **coefficients**	**Coefficients**	**Standardized** **coefficients**	**Coefficients**
CTQ → Adaptive CERQ	−0.27	−0.17^**^	−0.27	−0.17^**^
ASLEC → Maladaptive CERQ	0.43	0.15^***^	0.40	0.14^***^
ASLEC → Adaptive CERQ	−0.26	−0.12^*^	−0.26	−0.12^*^
CTQ → Maladaptive CERQ	−0.02	−0.01 ns		
Adaptive CERQ → NSSI	0.19	0.01^*^	0.23	0.01^*^
CTQ → NSSI	−0.23	−0.01^*^	−0.31	−0.01^***^
Maladaptive CERQ → HAMD	0.17	0.12^*^	0.17	0.12^*^
ASLEC → HAMD	0.31	0.08^**^	0.34	0.08^***^
Adaptive CERQ → HAMD	−0.08	−0.04 ns		
Maladaptive CERQ → NSSI	−0.13	−0.01 ns		
ASLEC → NSSI	−0.10	−0.00 ns		
CTQ → HAMD	0.19	0.07^*^	0.21	0.07^*^

*NSSI, non-suicidal self-injury; HAMD, Hamilton Rating Scale; CTQ, Childhood Trauma Questionnaire; ASLEC, Adolescent Self-Rating Life Events Checklist; CERQ, Cognitive Emotion Regulation Questionnaire; ns, non-significant.*

a *Final or modified model after removing non-significant paths.*

* *;p < 0.05.*

** *p < 0.01.*

*** *p < 0.001*.

**Figure 2:**
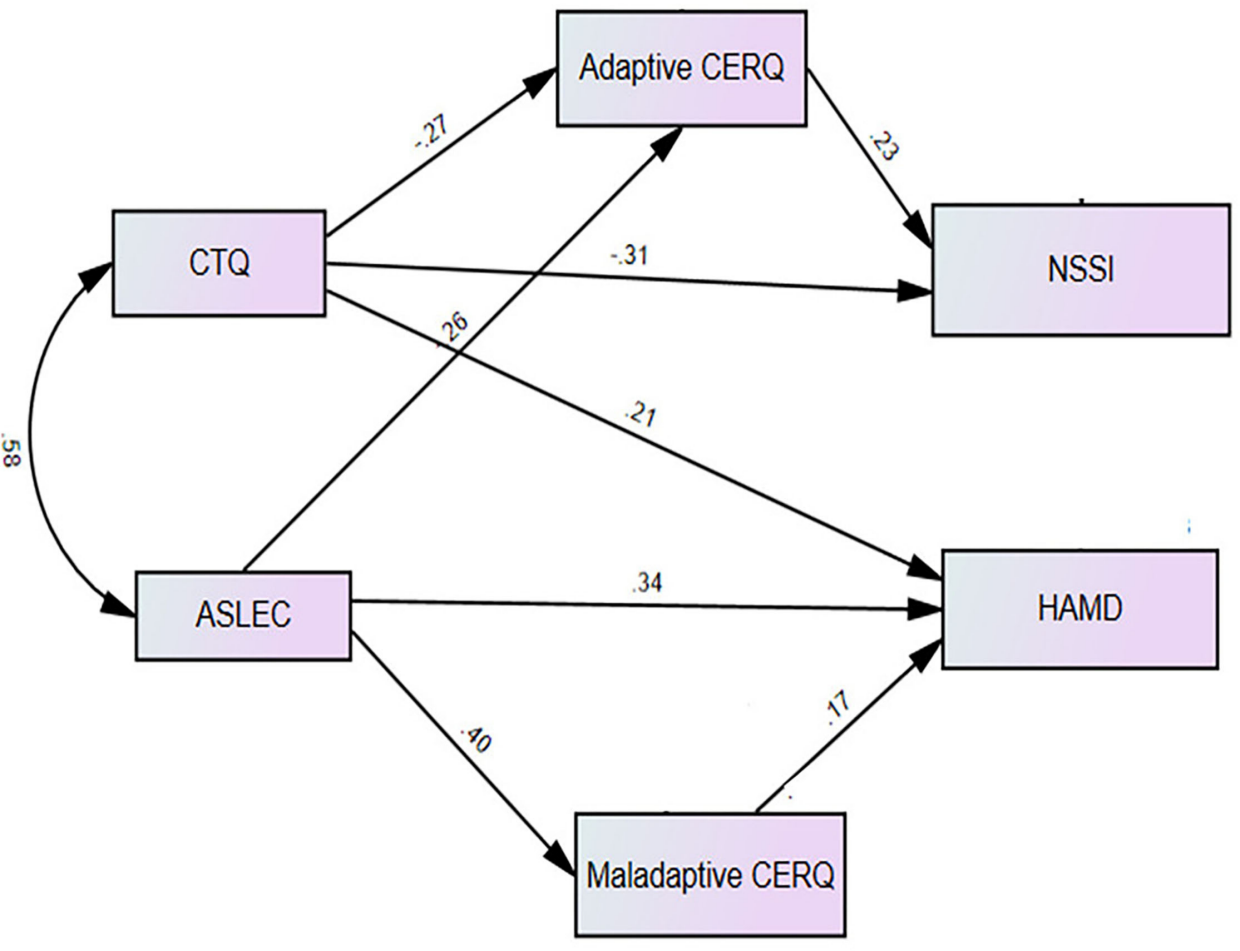
The final model, significant standardized path coefficients on the mediating role of adaptive and maladaptive CERQ.

The pathways from CM (β = −0.27, *p* < 0.01) and SLE (β = −0.26, *p* < 0.05) to adaptive CERS and the path from adaptive CERS to NSSI (β = 0.23, *p* < 0.05) were both significant. The direct path from CM to NSSI was significant (β = −0.31, *p* < 0.001), showing that adaptive CERS partially mediated the association between CM and NSSI. On the other hand, the direct path from SLE to NSSI was not significant, suggesting that adaptive CERS fully mediated the relationship between SLE and NSSI. Besides, the path from SLE to maladaptive CERS (β = 0.40, *p* < 0.001) and the path from maladaptive CERS to depression severity (β = 0.17, *p* < 0.05) were both significant. The direct path from SLE to depression severity was significant (β = 0.34, *p* < 0.001), showing that maladaptive CERS partially mediated the association between SLE and depression severity (Figure 2 and Table 4).

Table 5 summarizes bootstrapping results for the indirect effects of the final model. The results showed that indirect effects of CM (β = −0.006, 95% CI: −0.13, −0.008; *p* < 0.05), SLE (β = −0.006, 95% CI: −0.13, −0.002; p <0.05) on NSSI, mediated by adaptive CERS, were statistically significant. Thus, adaptive CERS partially mediated the association between CM and NSSI, but fully mediated the association between SLE and NSSI. CM and SLE had only direct effects on depression severity.

**Table 5:** The indirect effects in the final model using bootstrapping with 1,000 resamples.

**Paths**	**Standardized** **coefficients**	***p*-value**	**95% Bias corrected CI**	
			**Lower bound**	**Upper bound**
CTQ → Adaptive CERQ → NSSI	−0.06	0.02^*^	−0.13	−0.008
ASLEC → Adaptive CERQ → NSSI	−0.06	0.03^*^	−0.13	−0.002
ASLEC → Maladaptive CERQ → HAMD	0.07	0.06	−0.003	0.16

*NSSI, non-suicidal self-injury; HAMD, Hamilton Rating Scale; CTQ, Childhood Trauma Questionnaire; ASLEC, Adolescent Self-Rating Life Events Checklist; CERQ, Cognitive Emotion Regulation Questionnaire; CI, confidence interval.*

* *p < 0.05*.

## Discussion

The present study revealed that NSSI was highly prevalent in youth depression patients for approximately half of the total number of MDD. Adaptive, but not maladaptive, cognitive emotion regulation strategies affected the relationship of childhood maltreatment and stressful life events to NSSI. The direct effects of childhood maltreatment and stressful life events on depression severity were significant, but there were no indirect effects through cognitive emotion regulation strategies.

A primary aim of the current study was to broaden the data of the incidence of NSSI in youth MDD patients and identify the different clinical factors between MDD with and without NSSI. In our study, the frequency of NSSI was 49.1%. It showed higher than 16.9% of the youth (aged 14–21) who had ever harmed themselves (34) and 34.2% of undergraduate degree students (aged 18–26) with MDD who had a history of NSSI (8). Our results showed that the high rate of NSSI in youth MDD patients (aged 14–24), confirmed by previous studies that NSSI mostly occurs in adolescents and young adults (1, 34), and current depressive symptom can increase risk for NSSI and suicidal risk (35). In our study, no significant gender difference was shown between MDD patients with and without NSSI. Until now, the relationship between NSSI and gender is unclear. Many studies reported that women had a significant higher rate of NSSI than men in both clinical and community samples (36). The possible explanation was that the sample for this study was selected from adolescents and young adults of MDD patients with a high proportion of NSSI and female, which narrowed the sexual difference. In addition, this study differed from previous studies in the use of DSM-5 to define NSSI. We also found that those who have family history of mental illness showed more likely to injure themselves. Several prospective and cross-sectional studies suggested that parental depression may have a significant impact on the occurrence of NSSI in their children (19, 37, 38). A study (39) verified that individuals could produce NSSI behaviors by learning or imitating the behaviors of relatives and friends around them, which may, to some extent, explain the influence of family history of mental illness on NSSI. However, given that there is few evidence on this topic, further studies are needed to confirm the effect of family mental illness history on NSSI of children.

Compared with youth MDD patients without NSSI, youth MDD patients with NSSI were found to have more severity of depression, more experience of childhood maltreatment and stressful life events, more use of maladaptive cognitive emotion regulation strategies, and less use of adaptive cognitive emotion regulation strategies. These findings were consistent with previous studies. NSSI was an important risk factor for suicide in both psychiatric and nonclinical populations of adolescents and young adults (2). NSSI was also found to be associated with childhood maltreatment and stressful life events and the use of emotion regulation strategies (10, 11, 40). This study further confirmed that not only childhood maltreatment but also stressful life events (i.e., high academic pressure and tension with classmates or teachers) can also be a trigger factor of NSSI for youth with MDD. These findings underline the need for a broad clinical assessment of patients with depressive symptoms by including the assessment of childhood adversities, life events that occurred in adulthood, and emotion regulation strategies.

A secondary aim of this study was to examine direct and indirect links from childhood maltreatment and stressful life events to NSSI and depression severity *via* cognitive emotion regulation strategies. Our study indicates that the use of less overall adaptive cognitive emotion regulation strategies mediated the association from childhood maltreatment and stressful life events to NSSI among patients with greater childhood maltreatment experiences and stressful life events. A large number of studies have shown that childhood maltreatment cannot only directly affect NSSI but also indirectly affect NSSI through certain mediators (40). However, previous studies on the mediating effect of cognitive emotion regulation strategies from childhood maltreatment to NSSI were inconsistent in different populations. It had been reported that maladaptive cognitive emotion regulation strategies (self-blame) were identified as potential mediators between childhood maltreatment and NSSI in Australian adults (18). However, in psychiatric inpatient sample of youth, poor emotion expressivity, but not emotion coping, mediated the relation between childhood maltreatment and NSSI (7). Furthermore, emotion regulation strategies, which work adaptively for mentally healthy people, may produce maladaptive effects in clinical groups, leading to inconsistent adaptive strategy results. To our knowledge, there is limited studies that evaluated the mediated role of cognitive emotion regulation strategies between stressful life events and NSSI. This study was designed to focus on the population of adolescents and young adults with first-episode MDD and assessed the effects of childhood maltreatment and stressful life events on NSSI at the same time.

Therefore, it confirmed that adaptive cognitive emotion regulation strategies had a stronger mediating effect than maladaptive cognitive emotion regulation strategies. Our results highlight the ways in which childhood maltreatment and stressful life events create an environment that is chronically inhibiting children from developing adaptive emotional regulation strategies, and eventually lead to NSSI in youth MDD patients (41–43).

In our research, childhood maltreatment and stressful life events only had direct effects on depression severity. These findings were inconsistent with both maladaptive and adaptive strategies that mediated overall childhood maltreatment and depression severity (24), and maladaptive cognitive emotion regulation strategies were significant in the relationship between stressful life events and depressive symptoms (26). The possible reason for the discrepancy was that this study concurrently discussed the mediating model of cognitive emotion regulation strategies on NSSI and depression severity, while there was a considerable overlap between NSSI and depression (8, 44). Furthermore, cognitive emotion regulation strategies may have a stronger mediating effect on NSSI than depression severity, leading to the nonsignificant indirect pathway from childhood maltreatment and stressful life events to depression severity. Cognitive and metacognitive processes may explain why subjects with childhood maltreatment or stressful life events may show emotion dysregulation (45–48). A growing body of studies suggested that subjects with childhood adversities tend to engage in repetitive negative thinking (e.g., worry and rumination) and dysfunctional metacognitive processes (46, 47, 49), which, in turn, may lead to emotion dysregulation (45, 48). Furthermore, according to Hasking's cognition–emotion model theory of NSSI (21), early life stress may limit the development of emotion regulation strategies, then poor emotion regulation strategies may increase the risk of using NSSI as a form of emotion coping behavior.

The present study extends these findings by showing that the mediating role of adaptive emotion regulation strategies remains when childhood maltreatment and stressful life events are included. Furthermore, the unique role of adaptive emotion regulation strategies is worth noting. Both adaptive and maladaptive emotion regulation strategies are associated with NSSI in the current study, but only adaptive emotion regulation strategies emerge as a mediator. Our findings suggest that psychological interventions directed at childhood adversities and stressful life events may be included among psychological interventions for depressed patients. In addition, given that most people who seek treatment for depression experience relapse or recurrence, preventive psychological interventions after depression remission to prevent relapse and recurrence (6) in youth subjects with depressive symptoms exposed to stressful life events may be also considered. Literature shows that the sequential model (50, 51) may represent an efficacy approach for the management of recurrence and relapse in depression.

Our study has several limitations. First, the limitation of a cross-sectional study makes it impossible to infer causality in the childhood maltreatment and stressful life events on emotion regulation strategies and NSSI. Self-report questionnaire of stressful life events does not include its circumstances and consequences in detail to clarify the prevalence of dependent and independent stressful life events, which also make it questionable if this is a causative effect or merely the consequence of ill behavior. Second, due to the retrospective assessment of childhood maltreatment and stressful life events and the concurrent assessment of NSSI, it is uncertain that NSSI and child adversity did not overlap. Third, to explore psychosocial risk factors in MDD patients with NSSI, we only use MDD patients without NSSI as a control, but, healthy controls may be needed to determine whether there are differences between MDD patients and healthy people. Finally, in regard to the sample size, we only choose child adversity to evaluate the impact on NSSI, and additional research is needed to include a more comprehensive assessment of psychosocial factors (i.e., parenting style, family environment, peer relationship). Despite these limitations, the current study contributes to the existing literature on the understanding of NSSI in adolescents and young adult MDD patients and links between childhood maltreatment, stressful life events, emotion dysregulation, NSSI, and depression severity.

## Conclusions

First, this study found that the incidence of NSSI was high in youth MDD patients with no gender difference, more experience of childhood maltreatment, and stressful life events associated with the high rate of NSSI and more serious depression. Therefore, our study results draw attention to the importance of public health strategies to reduce the effect of childhood maltreatment and stressful life events. Second, this study demonstrated that adaptive cognitive emotion regulation strategies was an important possible mechanism underlying the negative effect of childhood maltreatment and stressful life events on NSSI in youth MDD patients. Early correction by strengthening adaptive cognitive emotion regulation strategies skills could be the effective prevention and intervention for NSSI of youth MDD patients with the experience of childhood maltreatment and stressful life events.

## Data Availability Statement

The raw data supporting the conclusions of this article will be made available by the authors, without undue reservation.

## Ethics Statement

The studies involving human participants were reviewed and approved by the Research Ethics Committee, Renmin Hospital of Wuhan University (Numbers: WDRY2020-K196). Written informed consent to participate in this study was provided by the participants' legal guardian/next of kin.

## Author Contributions

GW and HQ conceived the idea of the study and designed the research. GW, HQ, and CS analyzed the data and wrote the article. HQ, CS, LF, JX, and YG collected the data. All authors discussed the results and revised the manuscript. All authors contributed to the article and approved the submitted version.

## Conflict of Interest

The authors declare that the research was conducted in the absence of any commercial or financial relationships that could be construed as a potential conflict of interest.

## Publisher's Note

All claims expressed in this article are solely those of the authors and do not necessarily represent those of their affiliated organizations, or those of the publisher, the editors and the reviewers. Any product that may be evaluated in this article, or claim that may be made by its manufacturer, is not guaranteed or endorsed by the publisher.
